# Investigating interventions to increase uptake of HIV testing and linkage into care or prevention for male partners of pregnant women in antenatal clinics in Blantyre, Malawi: study protocol for a cluster randomised trial

**DOI:** 10.1186/s13063-017-2093-2

**Published:** 2017-07-24

**Authors:** Augustine T. Choko, Katherine Fielding, Nigel Stallard, Hendramoorthy Maheswaran, Aurelia Lepine, Nicola Desmond, Moses K. Kumwenda, Elizabeth L. Corbett

**Affiliations:** 1 Malawi-Liverpool-Wellcome Trust Clinical Research Programme, PO Box 30096, Chichiri, Blantyre 3, Malawi; 2 Liverpool School of Tropical Medicine, Liverpool, UK; 3 College of Medicine, Blantyre, Malawi; 4 London School of Hygiene and Tropical Medicine, London, UK; 5 Warwick Medical School, Coventry, UK

**Keywords:** Adaptive trials, HIV self-testing, Cluster randomised trials, HIV, Multi-arm multi-stage

## Abstract

**Background:**

Despite large-scale efforts to diagnose people living with HIV, 54% remain undiagnosed in sub-Saharan Africa. The gap in knowledge of HIV status and uptake of follow-on services remains wide with much lower rates of HIV testing among men compared to women. Here, we design a study to investigate the effect on uptake of HIV testing and linkage into care or prevention of partner-delivered HIV self-testing alone or with an additional intervention among male partners of pregnant women.

**Methods:**

A phase II, adaptive, multi-arm, multi-stage cluster randomised trial, randomising antenatal clinic (ANC) days to six different trial arms. Pregnant women accessing ANC in urban Malawi for the first time will be recruited into either the standard of care (SOC) arm (invitation letter to the male partner offering HIV testing) or one of five intervention arms offering oral HIV self-test kits. Three of the five intervention arms will additionally offer the male partner a financial incentive (fixed or lottery amount) conditional on linkage after self-testing with one arm testing phone call reminders.

Assuming that 25% of male partners link to care or prevention in the SOC arm, six clinic days, with a harmonic mean of 21 eligible participants, per arm will provide 80% power to detect a 0.15 absolute difference in the primary outcome. Cluster proportions will be analysed by a cluster summaries approach with adjustment for clustering and multiplicity.

**Discussion:**

This trial applies adaptive methods which are novel and efficient designs. The methodology and lessons learned here will be important as proof of concept of how to design and conduct similar studies in the future. Although small, this trial will potentially present good evidence on the type of effective interventions for improving linkage into ART or prevention. The trial results will also have important policy implications on how to implement HIVST targeting male partners of pregnant women who are accessing ANC for the first time while paying particular attention to safety concerns. Contamination may occur if women in the intervention arms share their self-test kits with women in the SOC arm.

**Trial registration:**

ISRCTN, ID: 18421340. Registered on 31 March 2016.

**Electronic supplementary material:**

The online version of this article (doi:10.1186/s13063-017-2093-2) contains supplementary material, which is available to authorized users.

## Background

Sub-Saharan Africa (SSA) accounts for 70% of the global HIV burden despite rapid scale up of HIV services including testing [1]. Analysis of the HIV care cascade indicates a striking fall-off in numbers between testing and linkage into HIV care or prevention [2]. Men regularly feature among populations with lower uptake of HIV testing across SSA [3] and lower rates of linkage into care or prevention [4] in the era of extremely ambitious targets for HIV [5]. The 90-90-90 targets aim to diagnose 90% of all HIV cases, start 90% of diagnosed HIV cases on treatment, and achieve viral suppression in 90% of those started on HIV treatment [5]. Awareness of HIV status among male partners of antenatal clinic (ANC) women attendees is low with less than 35% undergoing HIV testing when invited through their partner [6]. African women face substantial risk of acquiring HIV infection, estimated at 3.6% per pregnancy in study cohorts [7]. Over 90% of pregnant women access ANC services, providing an ideal opportunity to reach both partners with HIV testing and counselling services (HTS) [8].

A number of strategies have been found to increase uptake of HIV testing among male partners of ANC attendees, including home-based testing [9, 10], provider-initiated testing and counselling (PITC) [11], couples testing during antenatal visits [12] and home-based couple or partner testing [13, 14]. Key limitations of these strategies include: logistical difficulties of wide-scale implementation where home visits are required, lack of convenience, costs, lack of confidentiality and failure to prioritise men’s own health [15–17]. HIV self-testing (HIVST) is an alternative approach with the potential to increase couple or partner testing [18] and has been found to be highly acceptable to men in Malawi [19, 20]. Here, we define *HIVST-plus* as offering HIV self-testing along with an additional intervention aimed at improving linkage into HIV care or prevention. Such additional interventions include facilitated linkage [21], financial incentives (FI) [22] and short messaging services (SMS) [23].

This wide range of interventions presents technical challenges related to appropriate study design and analysis methods in order to identify optimal strategies [24]. Such complexity can be handled by applying multi-arm, multi-stage (MAMS) designs, which are more flexible by allowing pre-specified adaptations at interim analysis, as well as more efficient with respect to time and cost than standard parallel designs [25, 26]. In MAMS designs, several interventions are included in the first stage of the trial with pre-specified adaptations at interim analysis. Such a trial may either be a multi-stage phase II or III trial, or can be done as a seamless trial combining all the three trial phases separated by interim analyses [27]. In general, MAMS designs involve comparing each of several interventions to a control arm using interim analysis [25], providing an unbiased approach to investigating and selecting multiple phase II candidates under consideration for a future phase III trial [26]. Although predominantly used in the pharmaceutical industry to date, MAMS trial designs could have value in public health evaluations where randomising at the cluster level is often preferred. Furthermore, public health interventions are often complex involving multiple components and understanding the effect of each component may help inform the optimal choice [28].

Here, we describe the design of a phase II, adaptive, MAMS cluster randomised trial (CRT) with clinic day (not individual women) as the unit of randomisation. Our primary objective is to identify leading candidate interventions based on HIVST for improving HIV testing and linkage into care or prevention for male partners of ANC attendees in Blantyre, Malawi.

## Methods

### Design

**Fig. 1:**
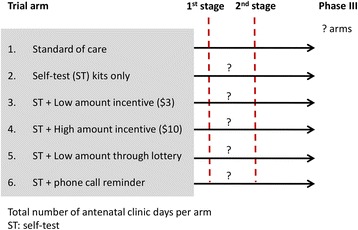
Schema of the phase II, adaptive, multi-arm, multi-stage cluster randomised trial. A two-stage, multi-arm, multi-stage (MAMS) trial design starting with six arms in the first stage. At interim analysis (end of first stage) some trial arms may be dropped, with recruitment to the remaining arms aiming to narrow down to a few arms that may be carried forward to a definitive (phase III) trial

This is a phase II, adaptive, MAMS CRT using ANC day as the unit of randomisation. As a phase II trial the study is intended to investigate *efficacy* relating to uptake of testing and subsequent HIV services by the male partner, *safety outcomes, and* to provide an estimate of *acceptability* to the pregnant woman. The trial will have one interim analysis during which pre-planned adaptations will be made as described below, followed by final analysis at the end of the second stage (two-stage MAMS design). The first stage will have six arms with one SOC and five intervention arms (Fig. 1). At the end of the first stage, a 3-point criteria will be considered by an independent Data Safety and Monitoring Board in order to drop intervention arms. Each intervention versus the SOC yielding *p* > 0.2 will be considered to be dropped; safety concerns; and costs will guide recommendations to drop or retain an intervention arm at the end of the first stage. The trial will not stop for efficacy at the interim analysis.

Analysis of second stage (end of trial) data will potentially lead to a definitive study (phase III) involving arms that show promise at the end of the second stage. A sample size of 36 ANC days (six per arm) will be required for the first stage (see the ‘Sample size considerations’ section below for justification). In order to control the family wise error rate (FWER) at the specified significance level (*α* = 0.2) for the five comparisons with the SOC arm, Dunnett’s test [29] will be applied.

### Trial simulation

A simulation study was set up to compute the overall probability of the trial to find at least one intervention whose efficacy is different compared to the SOC arm at the end of stage 2 (minimal power). The simulation was conducted under the alternative hypothesis with an assumed effect of 29–40% versus 25% in the SOC arm (Table 1). Additional key assumptions included that each ANC day (cluster) would have 20 participants with power set at 80% for each stage (Table 1). The simulation showed that at least two interventions would significantly improve the trial primary outcome (Table 2). These interventions would then be further tested in a potential phase III trial.

**Table 1:** Simulation inputs

Description	Input
Number of ANC days (clusters) per arm for both stages	6–20
Number of participants per ANC day	20
Linkage to care or prevention for the SOC arm in stages 1 and 2	25%
Linkage to care or prevention for the 5 intervention arms in stages 1 and 2	29–40%
SD of the mean of cluster-level proportions per arm (7 clusters, stage 1)^a^	0.05–0.08

*ANC* antenatal care, *SOC* standard of care, *SD* standard deviation

^a^Computed using *σ* = *k* × *μ* where *σ* is the standard deviation of the true cluster-level proportions; *k* is the coefficient of variation (assumed to be 0.2) and *μ* is the mean of the proportions per arm

Operating characteristics: stage 1 *α* = 0.2; stage 2 *α* = 0.1; 1 − *β* = 0.8

**Table 2:** Simulation results

	First stage		Simulations in which arm was dropped at interim^c^	Assumed
Study arm	^a^Proportion	SD^b^	Proportion	Proportion^d^
1 Standard of care	0.250	0.048	NA	0.250
2 Intervention	0.291	0.055	0.441	0.290
3 Intervention	0.299	0.058	0.336	0.300
4 Intervention	0.310	0.059	0.215	0.310
5 Intervention	0.321	0.061	0.114	0.320
6 Intervention	0.400	0.078	0.000	0.400

Exact intervention not specified here as there is no evidence about a particular one of the 5 interventions being investigated

*SD* standard deviation

^a^Proportion of male partners linked to care or prevention

^b^Assumed (0.050, 0.058, 0.060, 0.062, 0.064, 0.080) in arms 1–6, respectively

^c^Scenarios assumed for the 6 trial arms at the start

^d^If *p* value was > 0.2 at interim analysis, discontinued from recruitment

### Study setting and population

The study will recruit participants from Ndirande, Zingwangwa and Bangwe primary health centres (PHC) in urban Blantyre, Malawi. All women attending for antenatal care for the first time at these PHCs and their male partners will be eligible for participation (see Additional file 1). Women and their male partners will be excluded if they received couple or partner HIV testing in the current pregnancy; if either are aged below 18 years of age; if the male partner is reported to be HIV positive by the pregnant woman; if already recruited in this trial; and if not urban Blantyre resident. Malawi has recently (September 2016) implemented the test and treat approach where everyone diagnosed with HIV starts antiretroviral treatment (ART) immediately.

### Randomisation and recruitment flow

**Fig. 2:**
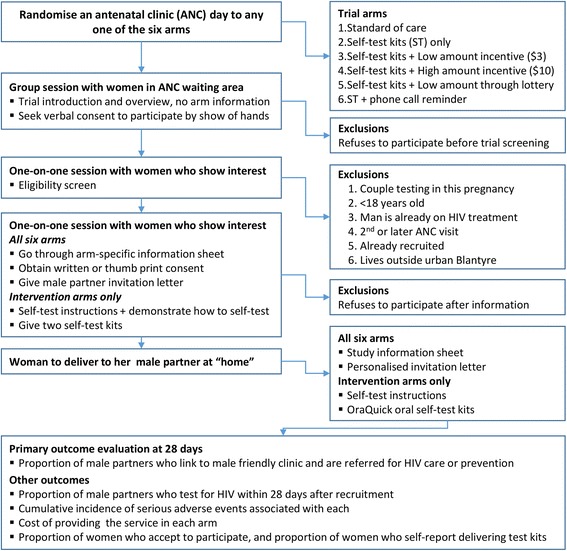
Randomisation, recruitment, and outcome evaluation. Each cluster (antenatal clinic day) is randomised to any of the six trial arms. All women attending their first antenatal clinic are briefed about the general purpose of the trial without receiving trial arm-specific information. Women then undergo one-on-one eligibility screen and arm-specific information. Women who are eligible and accept to participate are then given a male partner invitation letter alone or with two self-test kits to take home

Each ANC day was randomised to any one of the six trial arms using a randomised permuted block design in a ratio of 1:1:1:1:1:1 (Fig. 2). All three PHCs are of comparable size and serving comparable catchment populations, and, therefore, stratification was not deemed necessary. The allocation sequence was generated by an independent statistician using computer-generated random numbers [30]. The file containing the complete randomisation sequence will only be accessible to the independent statistician.

Field workers will enrol women into one of the six study arms on the morning of each ANC day after receiving the randomisation allocation for that ANC day (see Additional file 1). Women will receive study information in a group while in the ANC waiting area followed by one-on-one eligibility assessment and subsequent recruitment. Study information given during group sessions will not reveal details of arm-specific procedures to avoid potential non-participation associated with knowledge of procedures for each arm. All women who show interest at this stage will provide unwritten consent to participate by show of hands followed by written or witnessed thumb-print consent.

### Standard of care arm (SOC)

In the SOC arm women will receive a personalised letter only addressed to their male partner inviting him to go to the male-friendly clinic (MFC) to have an HIV test, receive HIV care or prevention and pregnancy-related education. The MFC is being implemented by the trial, and will offer men attending confirmatory HIV testing, facilitate linkage to HIV care or voluntary male medical circumcision (VMMC), and pregnancy-related health education.

### Intervention arms

In all five intervention arms, the woman will receive self-test instructions and two self-test kits to take home. The test kits, test instructions and a personalised letter will be delivered to the male partner by the woman in order to initiate dialogue for him to test and link to the MFC for HIV care or prevention as appropriate.

The five intervention arms differ with respect to financial incentives, participation in a lottery, and phone call reminders received by the male partner. Women in the first intervention arm will only receive the letter and the two self-test kits. In the two fixed financial incentive (FI) arms, male partners who self-test (test) and link into the MFC will receive an equivalent of US$3 or US$10 in the low- and high-FI arms, respectively. In the lottery FI arm, male partners who test and link into the MFC will have a 10% chance of winning US$30. In the final intervention arm, male partners will receive a phone call, through a number given to the study team by the woman at enrolment, to remind him to test and link into the MFC. All FIs will be disbursed as cash through mobile money in the trial in order to safeguard the safety of staff and are conditional on the male partner linking into the MFC.

### Primary and secondary outcomes

**Fig. 3:**
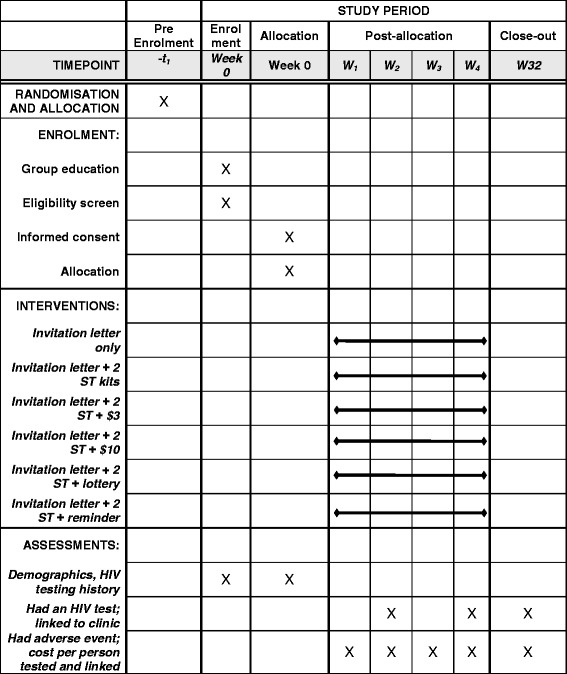
Schedule of enrolment, interventions and assessments (Standard Protocol Items: Recommendations for Interventional Trials (SPIRIT))

The primary outcome is the proportion of male partners of ANC attendees who test for HIV and link into HIV care or prevention within 28 days of enrolling the woman (Fig. 3). Thus, the primary outcome is defined as presentation of the male partner at the MFC with a used self-test kit (if in the intervention arm) or undergoing spot HIV testing with a study HIV counsellor within 28 days AND being referred for HIV care if HIV positive or VMMC if HIV negative and uncircumcised. There are four secondary outcomes: the proportion of male partners who test for HIV within 28 days (as reported by the woman); the proportion of women who accept to participate in their allocated trial arm; risk of serious adverse events (SAEs) in men and women in the study; and the total cost of implementing each trial arm. All outcomes will be analysed at cluster level (see the ‘Statistical analysis’ section).

### Outcome measurement

All male partners who present at the MFC in the SOC arm will be offered a single finger-prick HIV test with Determine 1/2™ as per Malawi national testing algorithm. An HTS counsellor will re-read a used self-test kit if the participant returns one as evidence of self-testing in the intervention arms. Participants who return with unused self-test kits or without self-test kits will be requested to self-test in the presence of the counsellor. All HIV results will be recorded on a data form followed by confirmation of HIV-positive results in parallel using Determine 1/2™ and Uni-Gold, with facilitated linkage to HIV care. All men who test HIV negative and report to be uncircumcised will be offered VMMC to be conducted by Population Services Internal (PSI). Thus, measurement of primary outcome includes evidence of an HIV test, confirmatory testing, and referral to HIV care or VMMC as appropriate within 28 days of the woman being recruited.

The secondary outcome of HIV testing among male partners will also be measured though proxy reporting by the woman using audio computer-assisted self-interview (ACASI) during her next ANC visit 4 weeks later. Participation in the allocated trial arm will be measured by computing the proportion of women who accept to participate after receiving trial-arm-specific information using the denominator of the total number of women who are eligible. All women will be asked to report any adverse events through ACASI at their next ANC visit while men who present to the MFC will be asked to report any adverse events. A costing tool validated in urban Blantyre [31] will be used to capture the costs associated with providing the service in each trial arm. The cost and outcome data will be used to estimate the cost per male partner tested for HIV, and cost per HIV-positive male identified through all SOC and intervention arms.

### Sample size considerations

A modification of the formula for sample size calculation for a MAMS design for binary outcomes [32] was made based on the methodology for CRTs [33] to identify each stage of the trial. We assume that each ANC day will have at least 40 women attending for the first time, 90% will satisfy the eligibility criteria and at least 60% will consent to participate, so having a cluster size of at least 21. We also assume that in the SOC arm 25% of men will satisfy the definition of the primary outcome [34]. For the first stage, six ANC days per arm (36 days in total) would be needed to detect an absolute difference of 15% in linkage compared to 25% in the SOC arm using a FWER of 0.2 with 80% pair-wise power and a coefficient of variation (*k*) of 0.10 [21]. In general, under the stated assumptions the trial has 80% chance of detecting a 1.6-fold increase in testing and linkage within 28 days compared to SOC at 5% significance level. Sample size for the second stage will be re-calculated based on empirical estimates at interim analysis with FWER of 0.1 and 80% power.

Very little clustering within ANC days is expected, hence we have assumed that *k* = 0.10 (intraclass correlation coefficient = 0.003). The simulation study assumed that *k* = 0.2 and differs with the final design in this respect resulting in six and seven clusters per arm in the final design and the simulation, respectively. A larger than conventional (0.05) FWER of 0.2 and 0.1 for stage 1 and stage 2, respectively, may lead to erroneously taking forward an ineffective intervention. However, given that this is a phase II trial this is not a major concern as it guards against dropping interventions that may otherwise prove to be effective in a larger, phase III trial. A more conventional pair-wise power of 0.8 was chosen to ensure that there is a high chance of taking forward most of the efficacious interventions from stage 1.

### Statistical analysis

Analyses will be done in R [35] and Stata 14.0 (Stata Corp., TX, USA). Baseline characteristics will be computed as proportions or median (interquartile range (IQR)), as appropriate, by arm in each of the two stages of the trial. Any variables that show imbalances will be adjusted [33] for when analysing the trial outcomes at the end of the second stage. We will assume that the two stages of the trial are independent [26] and will proceed to carry out a test of the null hypothesis of no difference in effectiveness of each intervention compared to the SOC. We will do this by analysing data from the first stage first followed by interim decisions to drop arms; then we will conduct and analyse data from the second stage (no overlap of participants from the first stage). Analysis of the whole trial will then be based on combined *p* values from both the first stage and the second stage using the weighted inverse normal (WIN) method [36] for arms that are not dropped at interim. A weighted average of the log (risk ratio (RR)) will be computed for the whole trial using estimates from each trial stage. All analyses will be by intention-to-treat taking as the denominator the number of women who were eligible and take into account the clustered design.

Given the small number (six) of clusters per arm in the first stage, analysis will be by cluster-level summaries using mean of proportion of male partners per clinic day who link to care or prevention in each arm [33]. The proportion of male partners who link into care or prevention will be computed per clinic day for each arm with number of men achieving the primary outcome and the number of women eligible and recruited in ANC on enrolment day as denominator. A log transformation of the clinic day proportions will be applied if a positive skew is observed [33]. The geometric mean of clinic day proportions in each of the five intervention arms will be compared to the SOC arm using an unpaired *t* test [33]. An estimate of the RR and a 95% CI will also be computed for each comparison by dividing the geometric mean of proportions in each intervention arm and the geometric mean of proportions in the SOC arm [33].

This analysis involves more than two comparisons with a single control arm which can lead to higher than the specified FWER or significance level. Therefore, Dunnett’s test [29] will be applied to the *t*-statistics generated from the unpaired *t* test to control the stage-wise FWER. Final decision-making at interim analysis will compare the Dunnett-corrected *p* values to stage 1 FWER of 0.2. The first of the three-part criteria for dropping trial arms will then be considered after examining final *p* values at the end of the first stage (interim analysis). Although sample size will be re-calculated at the end of stage 1, the total number of clinic days per arm is still presumed to be small for stage 2. Since the two stages are assumed to be independent, cluster-level summaries approach analogous to stage 1 analysis will also be followed in stage 2 comparing intervention arms that proceed to stage 2 with the SOC arm. A detailed analysis plan will be developed to guide analysis of the trial.

### Adaptations at interim analysis (end of stage 1)

Interim analysis at the end of stage 1 will assess whether any of the five intervention arms should be dropped as recommended by an independent Data Monitoring and Safety Board (DSMB) based on a three-part criteria. First, an arm whose statistical comparison to the SOC arm yields a *p* value > 0.2 will be considered for dropping for futility. Second, any intervention arm with a *high* incidence of SAEs, i.e. grades 3, 4 or 5 (Table 3) compared to SOC will be considered for dropping. It is at the discretion of the DSMB to decide, based on absolute number of SAEs in each intervention trial arm, whether they are high or not. Such an observation and recommendation will then be shared with the investigators who will make the final decision. Thirdly, an arm may be maintained after taking into account the costs associated with providing the service in light of the *p* value from statistical analysis. For this cost analysis, we will provide the DSMB estimates of the incremental cost per male partner tested, and incremental cost per HIV-positive male identified through the intervention arms in comparison to the SOC arm. The investigators will access the first-stage data only after the last follow-up visit for participants has occurred in order to perform interim analysis. The CRT extension to the Consolidated Standards of Reporting Trials (CONSORT) [37] will be followed when reporting the data.

**Table 3:** Adverse event grading

Grade 1 (Mild)	Grade 2 (Moderate)	Grade 3 (Severe) (within 30 days)	Grade 4 (Potentially life-threatening) (within 30 days)
1. Verbal, emotional or psychological intimate-partner violence (IPV) 2. Denying access to household resources 3. Being ignored 4. Being controlled (e.g. not allowed to leave house)	1. Coercion to self-test. 2. Coercion to disclose a self-test result 3. IPV that includes pushing, or slapping with an open hand that does not result in pain, or visible marks >24 h 4. Severe or prolonged psychological or emotional IPV leading to disruption of daily activities 5. Psychologically coercive sex	1. IPV that leads to pain, bruising or marks >24 hr 2. Threat of life-threatening violence (e.g. statement of intent to kill, mock strangulation, threatened with a knife or gun) 3. Physically coercive sex 4. Reports fearing for her life 5. Marriage break-up	1. IPV leading to hospitalisation or death 2. Suicide or attempted suicide 3. Attack using potentially lethal force (e.g. knife, gun, hammer, kicks to the head)

Grade 1 indicates a mild event

Grade 2 indicates a moderate event

Grade 3 indicates a severe event

Grade 4 indicates a potentially life-threatening event

Grade 5 indicates death: not indicated on the table

## Discussion

This is the first study that we are aware of that will use adaptive trial methodology in the context of randomising clusters rather than individuals. In this paper, we describe the methodological approaches to developing an adaptive CRT, to provide timely and cost-efficient understanding of optimal strategies to improve uptake of HIV testing and linkage into HIV care and prevention among male partners of pregnant women in a high HIV-prevalent setting. The methodology and lessons learned here will be important as proof of concept of how to design and conduct similar studies in the future. Being a phase II trial means that fewer resources can be allocated to a “learning” phase of a major phase III trial to narrow down to interventions that hold true rather than assumed potential effectiveness. As a multi-arm trial it allows the investigation of interventions that can act on their own, such as providing self-test kits only, or in combination, where an incentive or a reminder is given. This approach, which is one of the key strengths of adaptive trials, allows generation of clear evidence relating to specific intervention components that are effective when compared to the SOC.

The 2020 UNAIDS targets set in 2014 aim to diagnose 90% of people living with HIV and to start 90% of those diagnosed on ART, leading to virus suppression in 90% of those on ART [5]. While HIVST has been shown to increase uptake of HIV testing to within the first 90%, very limited evidence exists on effective interventions for improving linkage into care, the second 90%. In a recent trial in rural South Africa and Uganda, having a lay counsellor visit newly diagnosed individuals had minimal impact on linkage (risk ratio of 1.04) [38]. A combination strategy of conducting point-of-care CD4 at the time; accelerated ART initiation for adults with CD4 < 350 cells/uL; mobile phone appointment reminders; health educational packages; and non-cash financial incentives improved from 83% in the SOC arm to 92% in CRT in Swaziland [39]. However, the authors acknowledged that the multiplicity of interventions offered in the trial obscure the isolation of successful intervention components. Therefore, though small, this trial will potentially present good evidence on the type of effective interventions for improving linkage to HIV care or prevention, and also the right dose for financial incentives that may be effective.

The trial results will also have important policy implications on how to implement HIVST targeting male partners of pregnant women who are accessing ANC for the first time while paying particular attention to safety concerns. In a recent cohort study where HIV-negative pregnant women collected three oral self-test kits in Kenya 51% reported that their male partners had self-tested with none of the women reporting any SAEs [40]. Unlike in the Kenya study, where only HIV-negative pregnant women were eligible, this trial will recruit pregnant women, regardless of HIV status, who are attending their first ANC. The group of women recruited here receive the offer of an HIV test routinely making this trial design readily scalable. Measuring actual HIV testing is extremely difficult with HIVST as by definition disclosure depends on the individual. Our HIV testing outcome will be measured objectively through observed returned used/unused self-test kits by the man and also proxy reporting by the woman which minimises information bias.

A major anticipated constraint is potential for SAEs resulting mainly from intimate partner violence (IPV) to women, although evidence from studies using other populations and other HTS models suggests that this approach is unlikely to increase this problem [6]. A recent large HIV self-testing study in Malawi found no increase in IPV, despite an active community liaison system among 27,000 self-testing participants [20]. We will carefully monitor IPV, and have deliberately listed this as a secondary (safety) outcome. Although it will not be possible for participants and recruiting staff to predict the next-day recruiting arm, the knowledge of FI arms may result in altered decision-making about health care seeking. For example, a woman may choose to postpone her ANC attendance in the hope of being recruited in a FI arm, or indeed want to switch between arms.

There is potential for contamination if women in the intervention arms share their self-test kits with women in the SOC arm. In order to minimise this problem, we will ask women and their male partners in the intervention arms to bring used or unused self-test kits at follow-up and when they link into the MFC, respectively. We will also attempt to measure the magnitude of this problem by asking all women and male partners who link into the MFC in the SOC arm if they received self-test kits. There is potential bias in the estimation of the treatment effect and confidence intervals due to interim selection process of potential effective interventions which we will not explore.

## Trial status

At the time of submission on 9 November 2016, 32 of 36 clusters (total of 800 participants recruited) were covered for the first of the two trial stages. Interim analysis is planned for 20 January 2017. The second stage is planned to run for 4 months; we will compute the required sample size for the second stage at interim analysis to achieve the specified 80% power.

## Additional file

Additional file 1: SPIRIT Checklist. (DOC 151 kb)

## Abbreviations

ACASI: Audio computer-assisted self-interview
ANC: Antenatal clinic
ART: Antiretroviral treatment
CI: Confidence interval
COMREC: College of Medicine Research and Ethics Committee
CRT: Cluster randomised trial
DSMB: Data Safety and Monitoring Board
FI: Financial incentive
FWER: Family wise error rate
HIV: Human immunodeficiency virus
HIVST: HIV self-testing
HTS: HIV-testing services
IQR: Interquartile range
LSHTM: London School of Hygiene and Tropical Medicine
MAMS: Multi-arm multi-stage
MFC: Male-friendly clinic
MLW: Malawi Liverpool Wellcome Trust Clinical Research Programme
PHC: Primary health centre
PITC: Provider-initiated testing and counselling
RR: Risk ratio
SAEs: Serious adverse events
SD: Standard deviation
SMS: Short messaging service
SOC: Standard of care
SSA: Sub-Saharan Africa
VMMC: Voluntary male medical circumcision
WIN: Weighted inverse normal
