# Supplementary material for: Investigating interventions to increase uptake of HIV testing and linkage into care or prevention for male partners of pregnant women in antenatal clinics in Blantyre, Malawi: study protocol for a cluster randomised trial
Source: Trials. 2017 Jul 24;18:349. doi: 10.1186/s13063-017-2093-2 (PMC5525336; doi:10.1186/s13063-017-2093-2)
Supplement: Additional file 1: — SPIRIT Checklist. (DOC 151 kb) [file 13063_2017_2093_MOESM1_ESM.doc]

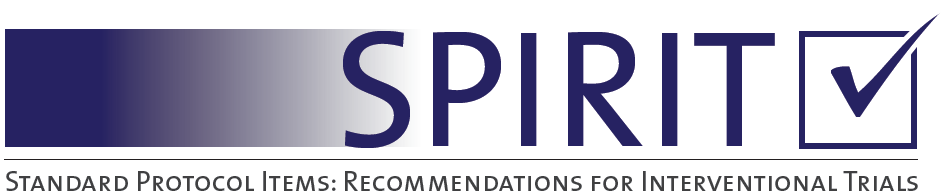


SPIRIT 2013 Checklist: Recommended items to address in a clinical trial protocol and related documents*

| Section/item | ItemNo | Description |
| --- | --- | --- |
| **Administrative information** | | |
| Title | 1 | **Effect of partner-provided HIV self-testing on HIV testing and linkage among male partners of antenatal clinic attendees in Malawi: study protocol for an adaptive Phase II cluster randomised trial (PASTAL)** |
| Trial registration | 2a | **Trial registry name:** ISRCTN. **Number:** ISRCTN18421340. **Date registered:** 31 Mar 2016 |
|  | 2b | See attached Table (Item 2b: SPIRIT.doc) |
| Protocol version | 3 | **Protocol:** version 1.5; 31 October 2016  **Revision chronology**  Version 1.2, 13 May 2016  Version 1.5, 31 October 2016  Primary reason for amendment: change in the definition of the primary outcome. |
| Funding | 4 | Funder: Wellcome Trust, UK |
| Roles and responsibilities | 5a | Augustine T. Choko*^1,4^, Katherine Fielding^4^, Nigel Stallard^5^, Hendramoorthy Maheswaran^5^, Aurelia Lepine^4^, Nicola Desmond^1,2^, Moses K. Kumwenda^1,3^, Elizabeth L. Corbett^1,3,4^   1. Malawi-Liverpool-Wellcome Trust Clinical Research Programme, Blantyre, Malawi 2. Liverpool School of Tropical Medicine, Liverpool, United Kingdom 3. College of Medicine, Blantyre, Malawi 4. London School of Hygiene & Tropical Medicine, London, United Kingdom 5. School of Public Health, Faculty of Health Sciences, University of the Witwatersrand, Johannesburg, South Africa 6. Warwick Medical School, Coventry, United Kingdom   **Corresponding author***  Malawi Liverpool Wellcome Trust Clinical Research Programme  P.O Box 30096  Chichiri  Blantyre 3  [augutc@gmail.com](mailto:augutc@gmail.com)  +265999577452  **Protocol contributors**  Grant holder: ATC  Conceived of the study: ATC and ELC  Provided statistical expertise in trial design: KF and NS  Provided expertise in interventional design: ATC, HM, AL, ND, MKK, and ELC  Implemented the trial: ATC and MKK  Conducting primary statistical analysis: ATC and KF  All authors contributed to refinement of the study protocol and approved the final manuscript. |
|  | 5b | The trial sponsor is London School of Hygiene & Tropical Medicine (Ref, No.: QA844), WC1E 7HT, Keppel Street, [+44 20 7636 8636](tel:%2B44 20 7636 8636), London, United Kingdom. Contact person: [patricia.henley@lshtm.ac.uk](mailto:patricia.henley@lshtm.ac.uk). |
|  | 5c | The funder and the sponsor played no role in the study design; collection, management, analysis, and interpretation of data; writing of the report; and the decision to submit the report for publication. |
|  | 5d | **Trial Steering Committee**   - Members: KF, ND, ELC. - Agreement of final protocol. - Recruitment of study participants. - Reviewing study progress and agreeing changes to the protocol if necessary. - Making final decision to drop arms following recommendation from Data Safety and Monitoring Board (DSMB) recommendation.   **Data Safety and Monitoring Board**   - Independent members from the trial investigators. Prof Frances Cowan (Chair), University College London; Prof Victor Mwapasa (College of Medicine, Malawi); and Dr Patrick Phillips (University College London and MRC, UK). - Reviewing interim analysis results including safety data. - Recommending that trial arms be dropped.   **Independent statistician**   - Dr Mavuto Mukaka (Wellcome Trust, Thailand). - Responsible for randomisation of trial units. |
| Introduction |  |  |
| Background and rationale | 6a | **Research question:** What are the most promising candidate interventions for increasing uptake of HIV testing and linkage into care or prevention for partners of pregnant women attending antenatal clinic in Blantyre, Malawi?  Sub-Saharan Africa (SSA) accounts for 70% of the global HIV burden despite rapid scale up of HIV services including testing. Analysis of the HIV care cascade indicates a striking fall-off in numbers between testing and linkage into HIV care or prevention. Men regularly feature among populations with lower uptake of HIV testing across SSA and lower rates of linkage into care or prevention in the era of extremely ambitious targets for HIV. The 90-90-90 targets aim to diagnose 90% of all HIV cases, start 90% of diagnosed HIV cases on treatment, and achieve viral suppression in 90% of those started on HIV treatment^5^. Awareness of HIV status amongst male partners of antenatal clinic (ANC) women attendees is low with less than 35% undergoing HIV testing when invited through their partner.  A number of strategies have been found to increase uptake of HIV testing among male partners of ANC attendees, including home-based testing, provider initiated testing and counselling (PITC), couples testing during antenatal visits and home-based couple or partner testing. Key limitations of these strategies include: logistical difficulties of wide scale implementation where home visits are required, lack of convenience, costs, lack of confidentiality and failure to prioritise men’s own health. HIV self-testing (HIVST) is an alternative approach with potential to increase couple or partner testing and has been found to be highly acceptable to men in Malawi. Here we define *HIVST-plus* as offering HIV self-testing along with an additional intervention aimed at improving linkage into HIV care or prevention. Such additional interventions include facilitated linkage, financial incentives (FI), and short messaging services (SMS). |
|  | 6b | **Comparator:** personalised male partner invitation letter given to the pregnant woman at enrolment at antenatal clinic |
| Objectives | 7 | Primary objective   1. To identify the most promising interventions for increasing both the uptake of HIV testing and linkage into HIV care or prevention among male partners of pregnant women attending ANC.   Secondary objectives   1. To identify the most promising interventions for increasing the uptake of HIV testing among male partners of pregnant women attending ANC. 2. To assess the acceptability of partner-provided HIVST-plus, as defined by willingness to deliver HIVST kits to male partners among women attending ANC. 3. To investigate the risk of intimate partner violence among women attending ANC who participate in the study. 4. To provide the cost associated with implementation of the service for each study arm |
| Trial design | 8 | This is a Phase II adaptive multi-arm multi-stage (MAMS) cluster randomised trial using antenatal (ANC) day as the unit of randomisation. Each ANC day was randomised to any one of the six trial arms using a randomised permuted block design in a ratio of 1:1:1:1:1:1. |
| Methods: Participants, interventions, and outcomes | | |
| Study setting | 9 | The study will recruit participants from Ndirande, Zingwangwa and Bangwe primary health clinics (PHC) in urban Blantyre, Malawi. |
| Eligibility criteria | 10 | **Inclusion criteria**   1. Women attending antenatal clinic for the first time at Ndirande, Zingwangwa and Bangwe PHC in urban Blantyre and their male partners. 2. ≥ 18 years old. 3. Have not had couple or partner testing in this pregnancy. 4. Male partner is unaware of being HIV positive. 5. Not already recruited in this trial. 6. Urban Blantyre resident.   **Exclusion criteria**   1. Have had couple or partner testing in this pregnancy. 2. <18 years old. 3. The man is already aware of their HIV positive status and receiving treatment. 4. Subsequent ANC visit 5. Already recruited in this trial. 6. Not present in urban Blantyre in the next days after the woman is enrolled in the trial. |
| Interventions | 11a | Intervention arm 1: Women receive a letter and two self-test kits to deliver to their male partners during their first antenatal clinic (ANC) visit.  Intervention arm 2: Women receive a letter and two self-test kits to deliver to their male partners who will get a financial incentive of $3 when they link into male friendly clinic and receive HIV care or HIV prevention services.  Intervention arm 3: Women receive a letter and two self-test kits to deliver to their male partners who will get an incentive of $10 when they link into male friendly clinic and receive HIV care or HIV prevention services.  Intervention arm 4: Women receive a letter and two self-test kits to deliver to their male partners who will be entered into a lottery with a 10% chance of winning $30 when they link into male friendly clinic and receive HIV care or HIV prevention services.  Intervention arm 5: Women receive a letter and two self-test kits to deliver to their male partners who will receive a phone call to remind them to test and link into male friendly clinic to receive HIV care or HIV prevention services. |
|  | 11b | Participants can withdraw at any time. |
|  | 11c | Participants are given trial information to improve participation in follow-up interviews with women four weeks after enrolment. |
|  | 11d | No drugs administered in the trial |
| Outcomes | 12 | ***Primary and secondary outcomes*** The primary outcome is the proportion of male partners of ANC attendees who test for HIV and link into HIV care or prevention within 28 days of enrolling the woman. Thus, the primary outcome is defined as presentation of the male partner at the MFC with a used self-test kit (if in the intervention arm) or undergoing on spot HIV testing with a study HIV counsellor within 28 days AND being referred for HIV care if HIV positive or VMMC if HIV negative and uncircumcised. There are four secondary outcomes: the proportion of male partners who test for HIV within 28 days; the proportion of women who accept to participate in their allocated trial arm; risk of serious adverse events (SAEs) by males and females in the study; and the total cost of implementing each trial arm. Exploratory outcome: a male partner with evidence of HIV testing within 28 days [either presenting with a used self-test kit or undergoing on spot HIV testing with a study HIV counsellor] AND being in pre-ART, received ART, received condoms, or undergone voluntary male medical circumcision (VMMC). |
| Participant timeline | 13 | Women are enrolled during their first antenatal clinic visit. Uptake of HIV testing and linkage to the trial-run male friendly clinic for HIV treatment if positive and voluntary male medical circumcision (VMMC) if negative are measured within 28 days among male partners of the pregnant women being recruited. Follow-up interviews are conducted with women four weeks after enrolment to measure safety outcomes and other outcomes. |
| Sample size | 14 | We assumed that each antenatal clinic day (cluster) will have at least 40 women attending for the first time, 90% would satisfy the eligibility criteria and at least 60% would consent to participate, so having a cluster-size of at least 21. We also assumed that in the standard of care (SOC) arm 25% of male partners will satisfy the definition of the primary outcome. For the first stage six antenatal clinic days per arm (36 days in total) would be needed to detect an absolute difference of 15% in linkage compared to 25% in the SOC arm using a family-wise error rate (FWER) of 0.2 with 80% pair-wise power and a coefficient of variation (k) of 0.10. Sample size for the second stage will be re-calculated based on empirical estimates at interim analysis with FWER of 0.1 and 80% power. |
| Recruitment | 15 | Strategies for achieving adequate participant enrolment to reach target sample size include group-based information while women wait for their antenatal care services followed by one-one information about each trial arm. |
| **Methods: Assignment of interventions (for controlled trials)** | | |
| Allocation: |  |  |
| Sequence generation | 16a | The allocation sequence was obtained using computer-generated random numbers with the three clinics as factors for stratification. Random permuted block randomisation was used to reduce predictability of a random sequence. |
| Allocation concealment mechanism | 16b | Telephone to the field team on the morning of recruitment was used as a mechanism of implementing the allocation sequence. |
| Implementation | 16c | Dr Mavuto Mukaka (Wellcome Trust, Thailand) was responsible for randomisation and intervention assignment of trial units. Field workers will enrol the participants. |
| Blinding (masking) | 17a | This is an unblinded trial although the trial investigators (outcome assessors) will only have aggregate data until the interim analysis. |
|  | 17b | The trial is unblinded |
| **Methods: Data collection, management, and analysis** | | |
| Data collection methods | 18a | All the data will be collected using open data kit (ODK) running on Nexus tablets. ***Outcome measurement*** All male partners who present at the male friendly clinic in the SOC arm will be offered a single finger prick HIV test with Determine 1/2™ as per Malawi national testing algorithm. An HTS counsellor will re-read a used self-test kit if the participant returns one as evidence of self-testing in the intervention arms. Participants who return with unused self-test kits or without self-test kits will be requested to self-test in the presence of the counsellor. All HIV results will be recorded on a data form followed by confirmation of HIV positive results in parallel using Determine 1/2™ and Uni-Gold, with facilitated linkage to HIV care. All men who test HIV negative and report to be uncircumcised will be offered VMMC to be conducted by Population Services Internal (PSI). Thus, measurement of primary outcome includes evidence of an HIV test, confirmatory testing, and referral to HIV care or VMMC as appropriate within 28 days of the woman being recruited.  The secondary outcome of HIV testing among male partners will also be measured though proxy reporting by the woman using audio computer-assisted self-interview (ACASI) during her next ANC visit four weeks later. Participation in the allocated trial arm will be measured by computing the proportion of women who accept to participate after receiving trial-arm specific information using the denominator of the total number of women who are eligible. All women will be asked to report any adverse events through ACASI at their next ANC visit while men who present to the MFC will be asked to report any adverse events. A costing tool validated in urban Blantyre^31^ will be used to capture the costs associated with providing the service in each trial arm. The cost and outcome data will be used to estimate the cost per male partner tested for HIV, and cost per male HIV-positive identified through all SOC and intervention arms. |
|  | 18b | In order to retain participants and to complete follow-up interviews with women we plan to:   - Check health passports of all women attending antenatal clinic (ANC) on days other than the first ANC. - Conduct telephone follow-up interviews for women with phone numbers who give prior consent during enrolment. - Inform male partners who link into the trial-run male friendly clinic to remind their partners about the follow-up visit.   Participants who discontinue from the trial will have a short version of the follow-up interview to measure trial outcomes. |
| Data management | 19 | Data will be managed through infrastructure set up within Malawi-Liverpool-Wellcome Trust Clinical Research Programme (MLW). Data collection and processing will be as detailed in the data management plan (DMP) included in Appendix R. Data will be collected using tablets running Open Data Kit (ODK) and will be downloaded onto a server running a MySQL Relational Database. Data quality assurance will be implemented within the electronic form so that out of range values, inconsistent values and required variables will be checked at the time of data collection. All tablets will have full log-in details of the person collecting the data including a password. Access to the study database will be protected by a password known only to the PI (Augustine Choko) and the IT systems administrator in MLW. Data for study monitoring will be periodically exported into comma separated values (CSV) from the study database on the MLW server for analysis and to raise plus resolve data queries.  Protocols for managing data without breach of confidentiality are in place within MLW. Access to the final data set will be limited to the PI (Augustine Choko), the Trial Steering Committee (TSC) and the Data Safety and Management Board (DSMB). Sensitive information (including HIV results) will not be linked to personal identifiers in the final data set. All devices and paper-based tools containing data will be kept in locked offices at MLW during data processing and in a locked data repository room for longer term storage. All data will be backed up daily by the MLW Data Office, with offsite back up once weekly. Backup data will be stored in a locked filing cabinet away from the office by the PI. |
| Statistical methods | 20a | Baseline characteristics will be computed as proportions or median (interquartile range [IQR]), as appropriate, by arm in each of the two stages of the trial. Any variables that show imbalances will be adjusted for when analysing the trial outcomes at the end of the second stage. All analyses will be by intention-to-treat taking as the denominator the number of women who were eligible and take into account the clustered design.  Given the small number (six) of clusters per arm in the first stage, analysis will be by cluster-level summaries using mean of proportion of male partners per clinic day who link to care or prevention in each arm. The proportion of male partners who link into care or prevention will be computed per clinic day for each arm with number of men achieving the primary outcome and the number of women eligible and recruited in ANC on enrolment day as denominator. A log transformation of the clinic day proportions will be applied if a positive skew is observed^33^. The harmonic mean of clinic day proportions in each of the five intervention arms will be compared to the SOC arm using unpaired t-test. An estimate of the risk ratio (RR) and a 95% CI will also be computed for each comparison.  The Dunnett test^29^ will be applied to the t-statistics generated from the unpaired t-test to control the stage-wise FWER. Final results will then be based on combined p-values from both the first stage and the second stage using the weighted inverse normal (WIN) method. |
|  | 20b | Any variables that show imbalances will be adjusted for when analysing the trial outcomes at the end of the second stage using logistic regression with random effects. |
|  | 20c | Analysis of the primary outcome will be by intention-to-treat. |
| **Methods: Monitoring** | | |
| Data monitoring | 21a | Data Safety and Monitoring Board (guided by Damocles Charter)   - Independent members from the trial investigators. Prof Frances Cowan (Chair), University College London; Prof Victor Mwapasa (College of Medicine, Malawi); and Dr Patrick Phillips (University College London and MRC, UK). - Reviewing interim analysis results including safety data. |
|  | 21b | Interim analysis at the end of stage 1 will assess whether any of the five intervention arms should be dropped as recommended by an independent data monitoring and safety board (DSMB) based on a 3-part criteria. First, an arm whose statistical comparison to the SOC arm yields a p-value>0.2 will be considered for dropping for futility. Second, any intervention arm with *high* incidence of SAEs i.e. grade 3, 4 or 5 (Table 1) compared to SOC will be considered for dropping. Thirdly, an arm may be maintained after taking into account the costs associated with providing the service in light of the p-value from statistical analysis. For this cost analysis, we will provide the DSMB estimates of the incremental cost per male partner tested, and incremental cost per male HIV positive identified through the intervention arms in comparison to the SOC arm. |
| Harms | 22 | There is potential for intimate partner violence (IPV) particularly to women although evidence from similar studies in Kenya suggest this approach is unlikely to increase this problem. All women will be asked through audio computer assisted self-interview (ACASI) if they experienced any adverse event including IPV four weeks after enrolment. Male partners who link into the trial-run male friendly clinic will also be asked to report if they experienced any adverse events particularly being coerced to have an HIV test. All adverse events will be captured and graded using tools developed as part of the trial. |
| Auditing | 23 | The trial will be independently audited according to standard operating procedures (SOPs) laid down by the Malawi Liverpool Wellcome Trust Clinical Research Programme (MLW) Clinical Trial Support Unit (CTSU) internal monitoring committee and the University of Malawi-College of Medicine Research Support Centre (RSC) monitoring process. |
| Ethics and dissemination | | |
| Research ethics approval | 24 | Ethics approval was obtained locally from the College of Medicine Research Ethics Committee (COMREC) in Malawi (approval number P.04/16/1932) and from the London School of Hygiene & Tropical Medicine Ethics Committee (approval number 11308). |
| Protocol amendments | 25 | Important protocol modifications (eg, changes to eligibility criteria, outcomes, analyses) will be communicated first to IRBs for approval before implementation. |
| Consent or assent | 26a | Field workers (data collectors) will obtain informed consent or assent from potential trial participants or authorised surrogates. All trial participants will give written or witnessed (with thumb print for illiterate participants) consent before undergoing any trial procedures. Written consent for male partners was waived by the two ethics committees because the first contact is with the woman. |
|  | 26b | Additional consent provisions for collection and use of participant data and biological specimens in ancillary studies (**not applicable**). |
| Confidentiality | 27 | Only authorised personnel will handle the study data with password protection of both the computer and the study database. Final data will be fully anonymised to remove any participant identifying information to uphold confidentiality. |
| Declaration of interests | 28 | All principal investigators declare no other competing interests for the overall trial and each study site. |
| Access to data | 29 | All trial investigators and DSMB members will have access to the final trial dataset. There are no contractual agreements that limit access for investigators. The final fully anonymised data from the study will be made publicly available through the LSHTM data repository (<http://datacompass.lshtm.ac.uk/>. |
| Ancillary and post-trial care | 30 | There are no ancillary and post-trial care, and for compensation to those who suffer harm from trial participation. |
| Dissemination policy | 31a | Findings from the trial will be disseminated to the Blantyre District Health Office (DHO) and officials in Ministry of Health (MoH) through presentations and final copy of the report. Further local dissemination will be done at the National AIDS Commission (NAC) / College of Medicine (COM) annual dissemination conference. Findings will also be presented at peer-reviewed regional and international conferences. Copies of the final report, published peer-reviewed paper (s) and abstracts will be made available to the COM Library, and to College of Medicine Research Ethics Committee (COMREC). |
|  | 31b | ICMJE authorship eligibility guidelines will be followed during publication. |
|  | 31c | The final fully anonymised data from the study will be made publicly available through the LSHTM data repository (<http://datacompass.lshtm.ac.uk/>.The trial registration number is ISRCTN18421340 and the full protocol can be shared with no restrictions on request. |
| Appendices |  |  |
| Informed consent materials | 32 | Model consent form and other related documentation given to participants and authorised surrogates (See attached Information sheet and consent form—**PQ23a**) |
| Biological specimens | 33 | Plans for collection, laboratory evaluation, and storage of biological specimens for genetic or molecular analysis in the current trial and for future use in ancillary studies, (**Not applicable**) |

*It is strongly recommended that this checklist be read in conjunction with the SPIRIT 2013 Explanation & Elaboration for important clarification on the items. Amendments to the protocol should be tracked and dated. The SPIRIT checklist is copyrighted by the SPIRIT Group under the Creative Commons “[Attribution-NonCommercial-NoDerivs 3.0 Unported](http://www.creativecommons.org/licenses/by-nc-nd/3.0/)” license.
